# Physiological Benchmarks and Player Profiling in Elite Football: A Role-Specific Analysis Using T-Scores

**DOI:** 10.3390/sports13060181

**Published:** 2025-06-10

**Authors:** Vincenzo Manzi, Daniele A. Cardinale, Marco Alfonso Perrone, Antonio Bovenzi, Ferdinando Iellamo, Cristian Savoia, Giuseppe Caminiti, Francesco Laterza

**Affiliations:** 1 Department of Education and Sports Sciences, Pegaso Open University, 80143 Naples, Italy; vincenzo.manzi@unipegaso.it (V.M.);; 2 Department of Physiology, Nutrition and Biomechanics, Åstrand Laboratory, The Swedish School of Sport and Health Sciences, 114 33 Stockholm, Sweden; daniele.cardinale@hotmail.com; 3 The Swedish Sports Confederation, 100 61 Stockholm, Sweden; 4 Department of Clinical Sciences and Translational Medicine, University of Rome Tor Vergata, 00133 Rome, Italy; iellamo@uniroma2.it; 5 Udinese Calcio, Serie A, 33100 Udine, Italy; antonio.bovenzi66@gmail.com; 6 The Research Institute for Sport and Exercise Sciences, The Tom Reilly Building, Liverpool John Moores University, Liverpool L3 5AH, UK; cristiansavoia@gmail.com; 7 Department of Human Science and Promotion of Quality of Life, San Raffaele Open University, 00166 Rome, Italy; giuseppe.caminiti@uniroma5.it; 8 Department of Neurosciences, Biomedicine and Movement Sciences, University of Verona, 37129 Verona, Italy

**Keywords:** maximal aerobic speed, anaerobic speed reserve, soccer, playing positions

## Abstract

Physiological characteristics such as VO_2max_, running economy (RE), maximal aerobic speed (MAS), maximal sprinting speed (MSS), anaerobic speed reserve (ASR), and player profiling (based on MSS and MAS) have been proven to be important for training prescriptions in football. However, previous studies on player profiling have neglected the absolute values of MSS and MAS. The objectives of this study were to compare the aforementioned physiological variables among player roles, create benchmarks, and provide normative data to help coaches categorize players, ultimately proposing a new player profiling method. We analyzed 195 male professional football players (50 forwards, 59 midfielders, 44 full-backs, and 42 center-backs). Multivariate analysis of variance with Tukey’s post hoc tests revealed positional differences. Center-backs exhibited lower VO_2max_ than midfielders and full-backs. Both center-backs and forwards showed poorer RE and MAS compared to midfielders and full-backs. Full-backs achieved higher MSS than midfielders and center-backs, and forwards outperformed center-backs. Finally, midfielders demonstrated lower ASR than all other positions. Benchmarks based on T-scores for all variables were provided. Finally, in the new profiling method proposed—also based on T-scores—players were classified as “speed”, “endurance”, or “hybrid” if their MAS and/or MSS T-score exceeded 60, “in development” if both were below 45, and “average” if both scores were between 45 and 60 without any value above 60. The normative data provided can assist coaches in identifying specific areas for improvement in players’ physical conditioning—particularly valuable for youth athletes or those returning from injury. Additionally, the new profiling method offers insights into individual player characteristics, enabling more tailored and effective training interventions.

## 1. Introduction

Football is a sport that requires an integrated combination of physical and technical–tactical parameters. The relative importance of each of these factors varies according to the specific needs of the game (tactical strategies of each team). From a physical point of view, football is characterized by a succession of explosive efforts over short distances (5–20 m) alternating with periods of low-intensity activity [1]. In modern football, among the game situations that require high physical intensity, pressing actions have assumed particular importance, especially during the transition phases following the loss of possession (counter-pressing) when a team aggressively tries to regain the ball in the advanced area of the pitch [2]. Although these actions have shown lower values in the main performance parameters, such as distance covered and speed reached, compared to other game phases, they are characterized by a high frequency of accelerations and decelerations. These stresses involve a large mechanical load and high energy expenditure, which strongly affects muscle glycogen depletion and neuromuscular fatigue in football players [2,3]. However, despite football involving frequent explosive efforts, aerobic metabolism represents the main energy source due to the long duration of the game [1]. In fact, the average intensity of the game is close to the anaerobic threshold, with values that generally oscillate between 80% and 90% of the maximum heart rate [4]. Kinematic analysis of the game has revealed how different roles require specific movement patterns, causing variations in locomotor activity [5].

In line with these findings, previous studies have also highlighted that players employed in roles where running is particularly important tend to show higher physical performance values [1,6,7]. To fully understand physical performance and design effective training programs, it is essential to differentiate between factors that depend on individual physiological determinants and those related to the tactical and motor demands of the player’s role on the pitch. For this reason, the evaluation of parameters such as maximum oxygen uptake (VO_2max_), running economy (RE), maximal sprinting speed (MSS), and anaerobic speed reserve (ASR) calculated as the difference between MSS and maximal aerobic speed (MAS), is of interest. These parameters directly influence a player’s ability to perform high-intensity actions repeatedly and provide possible practical indications to optimize training strategies.

The VO_2max_ is a key parameter for sustaining high aerobic workloads [8], and it has a significant impact on maximal aerobic speed (MAS). Cost of running, also known as running economy (RE), is defined as the metabolic cost or energy demand for a given velocity of running to cover a given distance [9]. RE can act as a compensatory mechanism: athletes with different VO_2max_ values can achieve similar endurance performance if the one with lower VO_2max_ has a better RE [10,11]. From a practical point of view, this means that players with a better RE can reach higher speeds with a lower energy expenditure. In this regard, Hoff and Helgerud estimated that a 5% improvement in RE can increase the distance covered in a match by about 1000 m [12]. In recent years, scientific research has highlighted the importance of the anaerobic speed reserve (ASR) as a relevant parameter to evaluate the ability of players to sustain high-intensity efforts beyond the MAS [13]. Some studies have highlighted that ASR can vary significantly even among players with the same role, reflecting individual physiological differences [14]. In particular, players with higher MSS values generally show a higher ASR [15]. In line with these findings, Ortiz et al. [14] demonstrated that ASR is also a strong predictor of players’ ability to perform intense accelerations and decelerations, which are fundamental actions in all roles. Moreover, ASR has also been proposed as a method to aid training prescriptions. In particular, higher volumes and lower intensities have been recommended for endurance-oriented runners, while lower volumes and higher intensities have been suggested for speed-oriented runners [16]. The ratio between MSS and MAS, known as the speed reserve ratio (SRR), has also been used to profile runners as having either more endurance or speed [17]. However, in the authors’ opinion, both methods (ASR and SRR) result in a loss of information, since they neglect the absolute values. For example, two players might both exhibit an ASR of 17 km/h—one with an MSS of 37 km/h and MAS of 20 km/h and another with an MSS of 33 km/h and MAS of 16 km/h—yet the former’s higher absolute values clearly reflect a substantially greater performance capacity. Therefore, a new approach might be necessary to address the problem.

Given the complex interaction between these variables and physical performance in football, defining reference values for key physiological parameters can provide essential tools to assess and improve individual player capabilities, as well as contribute to injury prevention. To date, however, no study has jointly analyzed VO_2max_, RE, MAS, MSS, and ASR within a cohort of elite football players. The lack of an integrated analysis limits the understanding of the interactions between these physiological parameters and their combined influence on specific performance in different roles. Therefore, the present study aimed to jointly analyze these physiological parameters in relation to different playing positions, with the aim of identifying any significant differences between roles. In addition, the second objective of this investigation was to provide benchmarks to the football practitioner community to facilitate monitoring and tailoring training strategies in line with the specific physical demands of modern football. Finally, a new profiling strategy will be presented.

## 2. Materials and Methods

### 2.1. Experimental Approach

This study was a retrospective analysis of existing data, aiming to examine the physiological and performance-related characteristics of professional football players across different playing positions, focusing on differences in running economy, aerobic power, and other key physiological parameters. Additionally, it aimed to establish normative data and benchmarks for evaluating football physiological performance in a rapid and intuitive manner. A cohort of elite-level football players competing in the Italian Serie A championship was monitored from the 2012–2013 to the 2023–2024 season, with data collected over twelve seasons from twelve different teams in the Italian First Division (Serie A). Determining the appropriate sample size for generating cohort-specific normative data is inherently complex; however, previous research indicates that a sample of 50 participants is generally adequate, while exceeding 75 does not yield additional benefits [18]. Given the well documented variations in physical performance based on playing position, players were categorized as follows: forwards and wingers (FWs), midfielders (MFs), full-backs (FBs), and center-backs (CBs). Goalkeepers were excluded from this study. Data that did not meet the inclusion criteria were removed, resulting in a final dataset of 195 valid entries. This included 50 forwards and wingers, 59 midfielders, 44 full-backs, and 42 center-backs, aligning with the recommendations of Bridges and Holler [18]. The variables analyzed were VO_2max_, MAS, RE, MSS, and ASR.

### 2.2. Subjects

The study was conducted during the football preseason period, specifically during the first two weeks of the preseason training camp leading up to the start of the Serie A Italian Football League. This research involved 195 male professional football players: 50 FWs (age 27.5 ± 3.7 years; height 182.4 ± 4.3 cm; body mass 79.9 ± 5.3 kg; fat mass 11.2 ± 1.9%), 59 MFs (age 28.0 ± 4.2 years; height 179.8 ± 4.7 cm; body mass 75.7 ± 4.9 kg; fat mass 10.5 ± 2.3%), 44 FBs (age 26.1 ± 3.2 years; height 181.2 ± 3.9 cm; body mass 77.6 ± 4.1 kg; fat mass 10.7 ± 2.0%), and 42 CBs (age 26.4 ± 3.8 years; height 189.5 ± 4.2 cm; body mass 84.1 ± 5.2 kg; fat mass 11.1 ± 1.8%). All athletes were active members of twelve different teams in the Serie A League, whose predominant playing formation was 4-3-3. Each athlete had a minimum of four years of experience at the highest competitive level. To improve the internal validity of this study, players were not aware of the research hypothesis underlying the study. The study was conducted in accordance with the ethical guidelines of the World Medical Association’s Code of Ethics and received approval from the Ethics Committee of the Tor Vergata University Hospital (protocol code 47.21). All subjects signed an informed consent. The study complied with the Declaration of Helsinki.

### 2.3. Fitness Assessment

#### 2.3.1. Maximal Oxygen Uptake (VO_2max_) Test

The players on separate occasions (24 h apart) performed two treadmill tests on a calibrated treadmill for VO_2max_ and RE assessment. On the first day, each participant underwent a VO_2max_ test to determine their maximal oxygen uptake. On the second day, they completed a RE test. The VO_2max_ test began with a 3 min warm-up at 8 km·h^−1^, followed by incremental increases of 1 km·h^−1^ every minute until voluntary exhaustion. Oxygen uptake was continuously measured using a portable metabolic system (K4b^2^, Cosmed, Rome, Italy), which employs a breath-by-breath method to capture real-time data with a measurement accuracy of 0.14 L/min and an intra-class correlation coefficient of 0.85 (test–retest reliability) [19], ensuring reliable data collection. Prior to testing, the system was calibrated for flow using a 3 L syringe and for gas composition using a standard mixture containing 16% O_2_ and 5% CO_2_ in nitrogen, ensuring measurement accuracy. The metabolic device was worn in a backpack to minimize any interference with running mechanics, and data were recorded and transmitted in real time to a computer for further analysis. Oxygen uptake values were averaged over 30 s intervals to reduce fluctuations and enhance data stability.

To confirm the attainment of VO_2max_, at least two of the following standard criteria had to be met [20]:

(a) A plateau in VO_2_ despite increasing speeds;
(b) A respiratory exchange ratio (RER) above 1.10;
(c) A heart rate (HR) within ±10 beats·min^−1^ of the age-predicted HRmax (208−0.7 × age) [21].

#### 2.3.2. Running Economy Test

On the second day, the running economy test was performed. The protocol began with a 6 min warm-up at 8 km·h^−1^, followed by 3 min of passive recovery. Subsequently, participants performed a 6 min running test at a fixed speed of 10 km·h^−1^ (~2.78 m/s), which corresponded to approximately 60% of their previously measured VO_2max_, followed by 3 min recovery and lactate sampling.

The energy cost of running (C_r_) was determined as the ratio of steady-state oxygen uptake (above resting levels) to running speed, expressed in meters per second [22,23]:

$$C_{r}=\frac{\dot{V}O_{2}-\dot{V}O_{2}\;(\mathrm{resting})}{\mathrm{Running}\;\mathrm{speed}\;(m/s)}$$

where

- $\dot{V}O_{2}$ is the measured oxygen uptake in ml O_2_·kg^−1^·min^−1^ during the test;
- $\dot{V}O_{2}$ (resting) is assumed to be 3.5 mL O_2_·kg^−1^·min^−1^;
- Running speed = 2.78 m/s (corresponding to 10 km/h).

This ratio, initially expressed as oxygen uptake per meter, was then converted into joules per kilogram per meter, using an energy equivalent of 20.9 J per liter of oxygen uptake:

$$Cr_{J}=Cr\;\times \;\frac{20.9}{1000}$$

This ensures standardized measurement units.

Blood lactate concentration was assessed from earlobe capillary blood samples (5 µL) collected using a portable amperometric microvolume lactate analyzer (Lactate Pro LT 1710, Arkray Inc., Kyoto, Japan). Before each test, the analyzer was calibrated following the manufacturer’s recommendations.

When blood lactate concentration ([La]) exceeded 2 mM·L^−1^, its contribution to C_r_ was determined by calculating the net increase in lactate (Δ[La]), as the difference between resting levels and the peak value observed during recovery:

$$\Delta [\mathrm{La}]=[\mathrm{La}{]}_{\mathrm{peak}}-[\mathrm{La}{]}_{\mathrm{resting}}$$

where resting blood lactate concentration was assumed to be 1 mM [24], (a commonly used reference value in physiological studies). The energetic contribution of lactate production was estimated using an energy equivalent of 60 J per kilogram per millimole of lactate produced [22] (corresponding to 3 mL O_2_·kg^−1^·mM^−1^):

$$E_{\mathrm{anaerobic}}=\Delta [\mathrm{La}]\;\times \;60$$

Blood samples were collected immediately after each step to assess its contribution to the energy cost of running, and again three minutes later to determine peak lactate concentration and estimate the anaerobic energy contribution. The RE was then obtained by dividing the anaerobic energy contribution by the total distance covered and adding it to the aerobic energy cost:

$$\mathrm{RE}=Cr_{J}+\frac{E_{\mathrm{anaerobic}}}{\mathrm{Total}\;\mathrm{distance}\;(m)}$$

This calculation integrates both aerobic and anaerobic contributions, providing a comprehensive assessment of the energy demand of running.

#### 2.3.3. Maximal Aerobic Speed (MAS) Calculation

The MAS represents the lowest running velocity at which an individual reaches VO_2max_. According to Di Prampero (1986) [25], MAS can be estimated as the ratio between VO_2max_ and RE:

$$\mathrm{MAS}=\frac{\dot{V}O2\max}{\mathrm{RE}}$$

where

- $\dot{V}O_{2\max}$ is the maximal oxygen uptake, measured in ml O_2_/kg/min, which can be converted to J/kg/s using an oxygen energy equivalent of 20.9 J per liter of O_2_ consumed;
- RE is the energy cost of running, expressed in J/kg/m after conversion from oxygen uptake.

Since $\dot{V}O_{2\max}$ is originally measured in ml O_2_/kg/min, it was first converted to J/kg/s using the relation [25]:

$$\dot{V}O2\max\left(J/\mathrm{kg}/s\right)=\dot{V}O2\max\left(\mathrm{ml}/\mathrm{kg}/\min\right)\;\times \;\frac{20.9}{1000}\;\times \;\frac{1}{60}$$

This transformation ensures that both VO_2max_ and RE are expressed in consistent units (J/kg/s and J/kg/m, respectively), allowing for a proper calculation of MAS in m/s. Once both parameters were obtained, MAS was calculated for each participant using this relationship. This value provides an individualized measure of aerobic performance and running efficiency, serving as a key reference for determining training intensities and evaluating endurance capacity.

#### 2.3.4. Maximal Sprint Speed (MSS) Assessment Procedure

Maximal Sprint Speed (MSS) was assessed using a 30 m linear sprint test with photocell gates, a widely recognized method for evaluating sprint performance in team sport athletes [26]. Before the test, participants completed a 10 min standardized warm-up, including dynamic stretching, mobility drills, progressive sprints, and neuromuscular activation exercises [27]. Each player began from a standing start position, ensuring one foot was placed 0.5 m behind the first timing gate to maintain consistency across trials. Sprint times were recorded using photocell gates (Brower Timing Systems, Salt Lake City, UT, USA; accuracy of 0.01 s) placed 0.4 m above the ground. Upon an auditory or visual cue, the athlete performed an all-out sprint along a 30 m track, with timing gates positioned at 0, 10, and 30 m [27]. The MSS was calculated as the highest speed attained over the final 20 m of the sprint, which has been validated as a reliable indicator of sprint performance [26,27]. Each athlete performed 3 maximal 30 m sprints, separated by 3 min of passive recovery to minimize fatigue effects [28]. 12The fastest recorded time was used to determine MSS, expressed in kilometers per hour (km/h).

#### 2.3.5. Anaerobic Speed Reserve (ASR)

Following MSS determination, this value was used to calculate the ASR, defined as the difference between MSS and MAS [13].

### 2.4. Benchmarks

To establish benchmarks for various physiological variables the T-score method was employed, and qualitative descriptions were assigned to facilitate interpretation. The T-score offers a more intuitive alternative to the z-score [29] and it is calculated as follows: (Z-score × 10) + 50, with a score of 50 rather than 0, equaling the mean. In agreement with previous studies [30,31], the categories were structured as follows: extremely poor (<20), very poor (≥20 to ≤30), poor (>30 to ≤40), below average (>40 to ≤45), average (>45 to ≤55), above average (>55 to ≤60), good (>60 to ≤70), very good (>70 to ≤80), and excellent (>80). Due to the different nature of ASR, the qualitative descriptors for this variable were extremely high, very high, high, above average, average, below average, low, very low, and extremely low. The benchmark method was also used to determine players’ profiles.

### 2.5. Statistical Analysis

Prior to conducting inferential analyses, a descriptive statistical analysis was performed for all dependent variables, including Running Economy (RE), Maximum Oxygen Uptake (VO_2max_), Maximum Aerobic Speed (MAS), Maximal Sprinting Speed (MSS), and Anaerobic Speed Reserve (ASR). Measures of central tendency and data dispersion were calculated (mean ± standard deviation). The Shapiro–Wilk test was applied to assess the normality of the data distribution, while Levene’s test was used to evaluate the homogeneity of variances across player positions. Variables that did not meet the assumption of normality were further examined through visual inspection of histograms and Q-Q plots. A Multivariate Analysis of Variance (MANOVA) was then conducted to evaluate the effect of player position on the dependent variables RE, VO_2max_, MAS, and MSS. MANOVA was chosen to account for potential correlations between these physiological variables and to reduce the risk of Type I errors associated with multiple separate tests. Pillai’s Trace was used as the multivariate test statistic, with statistical significance set at *p* < 0.05. Since the MANOVA revealed a significant effect of player position on the dependent variables (*p* < 0.05), separate one-way ANOVAs were performed for each variable (RE, VO_2max_, MAS, and MSS) to determine which specific variables exhibited significant differences among player positions. The significance level was set at *p* < 0.05 for all univariate analyses. For variables that showed significant differences in ANOVA, Tukey’s Honest Significant Difference (HSD) post hoc test was applied to identify which specific player positions differed from one another. This test was selected due to its ability to control the Type I error rate in multiple comparisons. Before performing MANOVA, a collinearity assessment was conducted using Pearson’s correlation matrix, Variance Inflation Factor (VIF), and the determinant of the correlation matrix. The analysis revealed that Anaerobic Speed Reserve (ASR) exhibited high collinearity with MAS and MSS, as indicated by an extremely low determinant value and an infinite VIF for some variables when ASR was included. Consequently, ASR was excluded from the MANOVA and analyzed separately using a univariate ANOVA to prevent multicollinearity from distorting the results. Effect sizes (ES) were calculated using Cohen’s d principle as follows: ES ≤ 0.2—trivial, 0.2< ES ≤ 0.6—small, 0.6 < ES ≤ 1.2—moderate, 1.2 < ES ≤ 2.0—large, 2.0 < ES ≤ 4.0—very large, and ES > 4.0—extremely large [32]. The analysis also incorporated two complementary methods to assess practical significance: the smallest worthwhile difference (SWD) and the smallest worthwhile change (SWC). Both approaches are grounded in Cohen’s effect size criterion of 0.2 [32]. All statistical analyses were conducted using Python (version 17.0.9) (statsmodels, scipy). The statistical significance threshold was set at *p* < 0.05 for all analyses.

## 3. Results

The mean, standard deviation, and smallest worthwhile change for VO_2max_, cost of running, maximal aerobic speed, maximal sprinting speed, and anaerobic sprint reserve across roles are shown in Table 1.

### 3.1. Role Comparison

The role-based comparison among playing positions revealed that center-backs exhibited lower values of VO_2max_ compared to midfielders and full-backs (ES = medium). Similarly, center-backs (ES = medium) and forwards (ES = large) demonstrated a worse RE than midfielders and full-backs. The same pattern was found for MAS, with both center-backs and forwards showing lower values than the other roles (ES = large). In terms of MSS, full-backs achieved greater speeds than midfielders and center-backs (ES = large), while forwards recorded higher speeds than center-backs with a medium effect size. Finally, for ASR, midfielders exhibited the lowest values, differing from forwards by a large effect size and from center-backs and full-backs by a medium effect size. All the role comparison data are presented in Table 2.

### 3.2. Benchmarks

T-score normative data and qualitative descriptions for each variable across playing position are shown in Table 3.

### 3.3. Players’ Profiling

**Figure 1:**
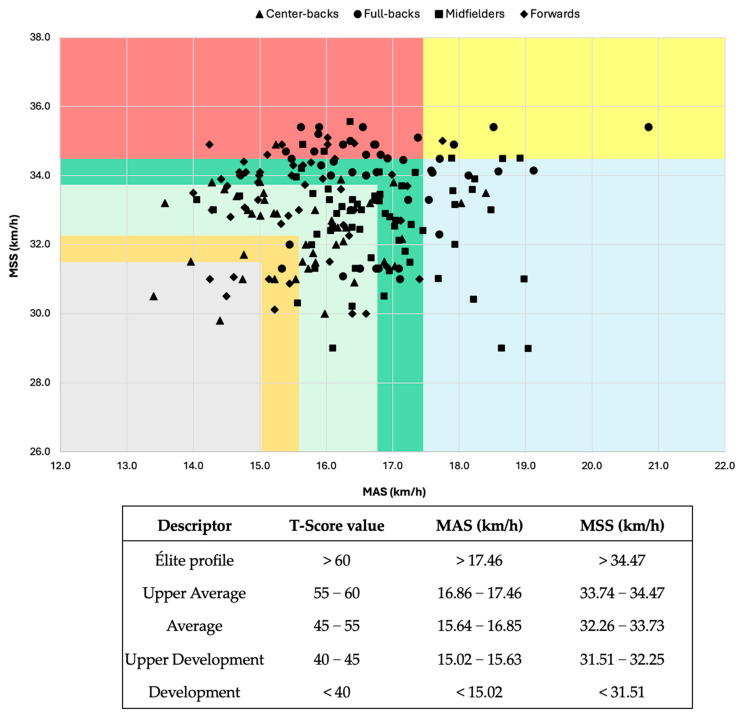
Players’ profiling. Graphical representation of player profiling based on MAS and MSS Values. MAS = maximal aerobic speed, MSS = maximal sprinting speed. Players are classified as “speed”, “endurance”, or “hybrid” if their MAS and/or MSS T-score exceeds 60, “in development” if both are below 45, and “average” if both are between 45 and 60 without any value above 60. Red = speed profile, yellow = hybrid, blue = endurance, dark green = upper average, light green = average, orange = upper development, and grey = development.

Players were categorized based on the benchmark method using absolute values of MAS and MSS. Specifically, players were classified as “speed”, “endurance”, or “hybrid” if their MAS and/or MSS exceeded a T-score of 60 (MAS > 17.46 km/h, MSS > 34.47 km/h). Those with both values below a T-score of 45 were labeled as “in development”, while players with T-scores between 45 and 60 were considered within the average range but were not assigned a specific profile due to the absence of high MAS or MSS values. A graphical representation of player profiling is provided in Figure 1. The percentage distribution of players classified as “speed”, “endurance”, or “hybrid” across different playing positions is reported in Table 4.

## 4. Discussion

The main findings of this investigation highlight how center-backs report lower VO_2max_, RE, MAS, and MSS. Full-backs report higher MSS and midfielders obtained greater values for MAS and VO_2max_ than the other roles. In the new profiling method proposed, players were classified as “speed”, “endurance”, or “hybrid” if their MAS and/or MSS T-score exceeded 60, “in development” if both were below 45, and “average” if both scores were between 45 and 60 without any value above 60. Finally, this is the first study on professional football players that provide benchmarks for all these variables at once: VO_2max_, MAS, RE, MSS, and ASR.

### 4.1. Role Comparison

In the literature, contrasting evidence has been reported regarding the difference among playing positions for physiological determinants. Same studies reported no significant differences in absolute VO_2max_ between different positions [33,34], while one study on Italian First Division players revealed that midfielders had the highest relative VO_2max_ [35]. Our findings partially confirmed the pattern observed by Paco et al. [35], as we reported lower VO_2max_ values for center-backs compared to midfielders and full-backs. This finding can be explained by the lower locomotor activity typically associated with the center-back role [5].

Midfielders and full-backs reported a better RE compared to center-backs and forwards, in agreement with Boone et al. [36]. This finding can be explained by the higher locomotor demands associated with these positions, which involve covering longer distances and performing a larger number of accelerations and decelerations during match play [37]. These movement patterns likely promote adaptations in neuromechanical and cardiorespiratory systems, leading to improved oxygen uptake efficiency and a reduced oxygen cost per unit of distance, thereby enhancing RE [38]. Contrary to previous studies, which found no difference in MAS among playing positions [34], our investigation showed that center-backs and forwards showed lower MAS than midfielders and full-backs. This result is partially consistent with study of Paco et al. [35], who found that maximal MAS was greater in midfielders than in forwards, with no differences among other roles. Regarding MSS, full-backs achieved higher speeds than both midfielders and center-backs, while forwards recorded higher speeds than center-backs. These results may be due to the fact that full-backs spend more time sprinting than players in other positions during matches [39]. The ASR between forwards, center-backs, and full-backs was comparable and statistically higher than the one exhibited by midfielders. This contrasts with the findings of Ortiz et al. [15], although it is fair to point out that in their study, players were categorized differently (as defenders, midfielders, and forwards).

### 4.2. Benchmarks

The second objective of this study was to compile normative data to help coaches evaluate players. The differences found among roles justify the role-based normative data and allow the analysis of players’ VO_2max_, MAS, RE, MSS, and ASR to understand if their performance is above or below average. This can help coaches to determine what capacity should be prioritized during training or, in terms of return-to-play, it can provide guidelines to understand when the player is ready to sustain the effort of the entire match. In addition, benchmarks may also be used to better understand when junior professional players have the same performance determinants of their more experienced teammates.

### 4.3. Players’ Profiling

In this study, we proposed a novel method for player profiling based on T-score values (>60) of MAS and MSS. This approach addresses the limitations of the speed reserve ratio (i.e., MSS/MAS) [17] by considering the absolute values of these variables. While the speed reserve ratio provides a straightforward means of assessing players, it may have limited practical utility if absolute values are not considered. The classification bands, illustrated in Figure 1, allow coaches to determine whether a player possesses a distinct physical profile. We argue that only players with a high MAS and/or MSS (T-score > 60) should be considered as having a specific profile, as their physical attributes warrant tailored training strategies.

For instance, a player with an MSS of 36 km/h is likely to spend more time at higher speeds compared to an average player with an MSS of 33 km/h under similar training conditions. Consequently, the player with a higher MSS will experience greater mechanical stress, requiring adjustments in both football training volume and sprint distance workload. Similarly, a player with a high MAS (e.g., 17 km/h) will likely tolerate and require higher training volumes to maintain aerobic capacity. Hybrid players—those exhibiting high values in both MSS and MAS—are particularly rare, with only 8 players out of 195 analyzed, and they can both sustain high training loads and reach high speeds. Conversely, players with average MAS and MSS values do not necessitate specific adjustments in training volume for the technical training, as their physiological characteristics do not impose unique demands that justify modifications.

The results presented in Table 4 highlight another important aspect of this investigation: 57.7% of players with a speed profile were full-backs, followed by forwards (26%), midfielders (11%), and center-backs (3.8%). The speed capacity of full-backs was also evident during match play, where this position distinguished itself by covering the greatest distance at very high-speed running [5]. Regarding the endurance profile, 66.7% of the players identified belonged to the midfielders, followed by full-backs (22.2%) and center-backs (11.1%). These findings are consistent with the physical demands typically associated with each role: full-backs often perform frequent high-speed runs, justifying their predominance in the speed profile group, while midfielders cover the greatest total distance during matches and engage in continuous submaximal efforts, which aligns with their strong representation in the endurance profile [13].

Finally, the hybrid profile was composed of 50% full-backs, 37.5% midfielders, and 12.5% forwards. This descriptive analysis suggests that speed and hybrid profiles are predominantly found among full-backs, while the endurance profile is most common in midfielders. Center-backs, who have previously been reported to exhibit lower locomotor activity [5,40], rarely possess any distinct profile. This further reinforces the idea that higher physiological capacities are not a primary requirement for this position, where tactical awareness may be the most crucial attribute.

## 5. Limitations

Some limitations should be considered when interpreting the results of this study. Since the VO_2max_ and the energy cost of running were measured using a portable metabolic system, caution is needed when comparing these values to those estimated from field tests conducted without a metabolic system. Second, given the potential differences in activity profiles across leagues, caution is warranted when applying these results to other championships [41]. It is important to note that in this investigation, RE was measured at a submaximal speed of 10 km/h; therefore, caution is warranted when comparing these values to RE measurements taken at higher running speeds, as they may reflect different physiological responses.

## 6. Conclusions

This study provides valuable insights into the physiological characteristics of professional football players across different playing positions in the Italian Serie A. Our findings underscore the existence of distinct physiological profiles linked to specific playing positions, suggesting that training and tactical approaches should be tailored accordingly. By establishing benchmarks and normative data on VO_2max_, RE, MAS, MSS, and ASR, coaches and practitioners are equipped with practical tools to assess and categorize athletes more effectively. These metrics can be used as a comparative method to evaluate the readiness of young professionals transitioning to elite levels and to set performance goals for experienced players returning from injury. Moreover, the smallest worthwhile changes provided may help identify meaningful changes and assess the effectiveness of training programs.

The proposed T-score-based profiling method offers a more nuanced classification system that overcomes previous limitations by incorporating absolute values. This new categorization allows for more precise evaluation and tailored training prescriptions. Overall, the framework can support individualized training programs, player development monitoring, and return-to-play decisions. Future research should explore the application of this profiling method across different leagues and levels of play to enhance its generalizability. Additionally, comparing these parameters before and after matches may offer valuable insights into how fatigue impacts a player’s ability to perform and could open new perspectives on resilience as a potential skill to be considered.

## Figures and Tables

**Table 1:** Mean, standard deviation, and smallest worthwhile change for VO_2max_, running economy, maximal aerobic speed, maximal sprinting speed, and anaerobic sprint reserve across playing roles.

Position	VO_2max_ (mL/kg/min)	SWC (mL/kg/min)	RE (J/kg/m)	SWC (J/kg/m)	MAS (km/h)	SWC (km/h)	MSS (km/h)	SWC (km/h)	ASR (km/h)	SWC (km/h)
Center Back	55.12 ± 3.57	0.714	4.43 ± 0.252	0.050	15.62 ± 1.08	0.215	32.38 ± 1.16	0.232	16.76 ± 1.51	0.303
Full-back	57.53 ± 3.60	0.721	4.28 ± 0.238	0.048	16.87 ± 1.08	0.216	33.85 ± 1.38	0.276	16.98 ± 1.63	0.327
Midfielder	57.67 ± 4.05	0.810	4.30 ± 0.241	0.048	16.84 ± 1.05	0.210	32.63 ± 1.46	0.292	15.78 ± 1.94	0.388
Forward	56.40 ± 3.71	0.742	4.56 ± 0.260	0.052	15.53 ± 0.93	0.186	33.16 ± 1.47	0.294	17.62 ± 1.79	0.357

SWC = smallest worthwhile change, RE = running economy, MAS = maximal aerobic speed, MSS = maximal sprinting speed, ASR = anaerobic sprint reserve.

**Table 2:** Role-based comparison of each variable among playing positions.

VO_2max_		Mean Difference	*p*-Value	Cohen’s d	Quantitative Relevance	SWD	Practical Relevance
Center-backs vs.	Forwards	−1.28	0.371	−0.339	Small	0.351	Moderate
	Midfielders	−2.55	0.005 *	−0.676	Medium	0.661	Meaningful
	Full-backs	−2.41	0.017 *	−0.641	Medium	0.672	Meaningful
Forwards vs.	Midfielders	−1.27	0.298	−0.338	Small	0.326	Moderate
	Full-backs	−1.14	0.462	−0.302	Small	0.309	Moderate
Midfielders vs.	Full-backs	0.13	0.998	0.035	Negligible	0.036	Negligible
**RE (J/kg/min)**		**Mean Difference**	***p*-value**	**Cohen’s d**	**Quantitative relevance**	**SWD**	**Practical Relevance**
Center-backs vs.	Forwards	−0.127	0.074	−0.510	Medium	0.507	Moderate
	Midfielders	0.134	0.040 *	0.541	Medium	0.529	Moderate
	Full-backs	0.150	0.029 *	0.603	Medium	0.612	Moderate to High
Forwards vs.	Midfielders	0.261	<0.001 ^†^	1 051	Large	1.040	High
	Full-backs	0.276	<0.001 ^†^	1 114	Large	1.120	High
Midfielders vs.	Full-backs	0.015	0.989	0.062	Negligible	0.083	Negligible
**MAS (km/h)**		**Mean Difference**	***p*-value**	**Cohen’s d**	**Quantitative relevance**	**SWD**	**Practical Relevance**
Center-backs vs.	Forwards	0.090	0.975	0.087	Negligible	0.090	Negligible
	Midfielders	−1.219	<0.001 ^†^	−1 178	Large	1.148	Large
	Full-backs	−1.248	<0.001 ^†^	−1 206	Large	1.157	Large
Forwards vs.	Midfielders	−1.309	<0.001 ^†^	−1 266	Large	1.314	Large
	Full-backs	−1.338	<0.001 ^†^	−1 294	Large	1.336	Large
Midfielders vs.	Full-backs	−0.029	0.999	−0.028	Negligible	0.028	Negligible
**MSS (km/h)**		**Mean Difference**	***p*-value**	**Cohen’s d**	**Quantitative relevance**	**SWD**	**Practical Relevance**
Center-backs vs.	Forwards	−0.779	0.039 *	−0.562	Medium	0.583	Moderate
	Midfielders	−0.246	0.816	−0.177	Negligible	0.186	Negligible
	Full-backs	−1.475	<0.001 ^†^	−1 065	Large	1.151	High
Forwards vs.	Midfielders	0.533	0.191	0.385	Small	0.362	Moderate
	Full-backs	−0.696	0.075	−0.502	Medium	0.483	Moderate
Midfielders vs.	Full-backs	−1.229	<0.001 ^†^	−0.887	Large	0.855	High
**ASR (km/h)**		**Mean Difference**	***p*-value**	**Cohen’s d**	**Quantitative relevance**	**SWD**	**Practical Relevance**
Center-backs vs.	Forwards	−0.869	0.088	−0.494	Small	0.516	Moderate
	Midfielders	0.946	0.042 *	0.537	Medium	0.552	Moderate
	Full-backs	−0.227	0.932	−0.129	Negligible	0.140	Negligible
Forwards vs.	Midfielders	1.815	<0.001 ^†^	1 032	Large	0.982	High
	Full-backs	0.642	0.293	0.365	Small	0.373	Moderate
Midfielders vs.	Full-backs	−1.173	0.005 *	−0.667	Medium	0.661	Moderate to High

RE = running economy, MAS = maximal aerobic speed, MSS = maximal sprinting speed, ASR = anaerobic sprint reserve, * = *p* < 0.05, ^†^ = *p* < 0.01.

**Table 3:** T-score normative data for each variable across playing positions.

VO_2max_ (mL/kg/min)	T-Score Value	Center-Backs	Full-Backs	Midfielders	Strikers
Excellent	>80	>65.83	>68.35	>69.94	>67.53
Very good	70–80	62.26–65.83	64.74–68.35	65.81–69.94	63.82–67.53
Good	60–70	58.69–62.26	61.14–64.74	61.69–65.81	60.11–63.82
Above Average	55–60	56.91–58.69	59.34–61.14	59.63–61.69	58.25–60.11
Average	45–55	53.34–56.91	55.73–59.34	55.51–59.63	54.54–58.25
Below average	40–45	51.55–53.34	53.93–55.73	53.45–55.51	52.69–54.54
Poor	30–40	47.98–51.55	50.33–53.93	49.32–53.45	48.98–52.69
Very poor	20–30	44.42–47.98	46.73–50.33	45.20–49.32	45.27–48.98
Extremely poor	<20	<44.42	<46.73	<45.20	<45.27
**RE (J/kg/min)**	**T-Score value**	**Center-backs**	**Full-backs**	**Midfielders**	**Strikers**
Excellent	>80	<3.68	<3.57	<3.57	<3.78
Very good	70–80	3.93–3.68	3.81–3.57	3.82–3.57	4.04–3.78
Good	60–70	4.18–3.93	4.05–3.81	4.06–3.82	4.30–4.04
Above Average	55–60	4.31–4.18	4.16–4.05	4.18–4.06	4.43–4.30
Average	45–55	4.56–4.31	4.40–4.16	4.42–4.18	4.69–4.43
Below average	40–45	4.68–4.56	4.52–4.40	4.54–4.42	4.82–4.69
Poor	30–40	4.94–4.68	4.76–4.52	4.78–4.54	5.08–4.82
Very poor	20–30	5.19–4.94	5.00–4.76	5.02–4.78	5.34–5.08
Extremely poor	<20	>5.19	>5.00	>5.02	>5.34
**MAS (km/h)**	**T-Score value**	**Center-backs**	**Full-backs**	**Midfielders**	**Strikers**
Excellent	>80	>18.87	>20.11	>20.12	>18.32
Very good	70–80	17.79–18.87	19.03–20.11	19.02–20.12	17.39–18.32
Good	60–70	16.70–17.79	17.95–19.03	17.92–19.02	16.46–17.39
Above Average	55–60	16.16–16.70	16.87–17.95	17.37–17.92	16.00–16.46
Average	45–55	15.08–16.16	16.33–16.87	16.26–17.37	15.07–16.00
Below average	40–45	14.54–15.08	15.79–16.33	15.71–16.26	14.60–15.07
Poor	30–40	13.46–14.54	14.71–15.79	14.61–15.71	13.68–14.60
Very poor	20–30	12.38–13.46	13.63–14.71	13.51–14.61	12.75–13.68
Extremely poor	<20	<12.38	<13.63	<13.51	<12.75
**MSS (km/h)**	**T-Score value**	**Center-backs**	**Full-backs**	**Midfielders**	**Strikers**
Excellent	>80	>35.86	>38.00	>37.00	>37.57
Very good	70–80	34.70–35.86	36.62–38.00	35.54–37.00	36.10–37.57
Good	60–70	33.54–34.70	35.24–36.62	34.08–35.54	34.63–36.10
Above Average	55–60	32.96–33.54	34.54–35.24	33.35–34.08	33.89–34.63
Average	45–55	31.80–32.96	33.16–34.54	31.89–33.35	32.42–33.89
Below average	40–45	31.22–31.80	32.47–33.16	31.16–31.89	31.69–32.42
Poor	30–40	30.06–31.22	31.09–32.47	29.70–31.16	30.22–31.69
Very poor	20–30	28.90–30.06	29.71–31.09	28.24–29.70	28.75–30.22
Extremely poor	<20	<28.90	<29.71	<28.24	<28.75
**ASR (km/h)**	**T-Score value**	**Center-backs**	**Full-backs**	**Midfielders**	**Strikers**
Extremely high	>80	>21.30	>21.88	>21.74	>22.99
Very high	70–80	19.78–21.30	20.25–21.88	19.76–21.74	21.20–22.99
High	60–70	18.27–19.78	18.62–20.25	17.79–19.76	19.41–21.20
Above Average	55–60	17.51–18.27	17.80–18.62	16.80–17.79	18.52–19.41
Average	45–55	16.00–17.51	16.17–17.80	14.82–16.80	16.73–18.52
Below average	40–45	15.24–16.00	15.35–16.17	13.83–14.82	15.84–16.73
Low	30–40	13.73–15.24	13.72–15.35	11.86–13.83	14.05–15.84
Very low	20–30	12.22–13.73	12.09–13.72	9.88–11.86	12.27–14.05
Extremely low	<20	<12.22	<12.09	<9.88	<12.27

RE = running economy, MAS = maximal aerobic speed, MSS = maximal sprinting speed, ASR = anaerobic sprint reserve.

**Table 4:** Distribution of player profiles across playing positions.

Player Profile	Role			
	Forwards (%)	Full-Backs (%)	Center-Backs (%)	Midfielders (%)
Speed (n = 26)	26.9	57.7	3.8	11.5
Endurance (n = 18)	0.0	22.2	11.1	66.7
Hybrid (n = 8)	12.5	50.0	0.0	37.5

## Data Availability

Data are available upon request from the corresponding author due to privacy and ethical restrictions.

## Abbreviations

VO_2max_	maximal oxygen uptake
RE	running economy
MAS	maximal aerobic speed
MSS	maximal sprinting speed
ASR	anaerobic speed reserve
FWs	forwards
FBs	full-backs
MFs	midfielders
CBs	center-backs
ES	effect size
